# Association Study of Gut Flora in Coronary Heart Disease through High-Throughput Sequencing

**DOI:** 10.1155/2017/3796359

**Published:** 2017-04-09

**Authors:** Li Cui, Tingting Zhao, Haibing Hu, Wen Zhang, Xiuguo Hua

**Affiliations:** ^1^Key Laboratory of Veterinary Biotechnology, School of Agriculture and Biology, Shanghai Jiao Tong University, Shanghai 200240, China; ^2^School of Medical Science and Laboratory Medicine, Jiangsu University, Jiangsu 212013, China

## Abstract

*Objectives.* We aimed to explore the impact of gut microbiota in coronary heart disease (CHD) patients through high-throughput sequencing.* Methods.* A total of 29 CHD in-hospital patients and 35 healthy volunteers as controls were included. Nucleic acids were extracted from fecal samples, followed by *α* diversity and principal coordinate analysis (PCoA). Based on unweighted UniFrac distance matrices, unweighted-pair group method with arithmetic mean (UPGMA) trees were created.* Results.* After data optimization, an average of 121312 ± 19293 reads in CHD patients and 234372 ± 108725 reads in controls was obtained. Reads corresponding to 38 phyla, 90 classes, and 584 genera were detected in CHD patients, whereas 40 phyla, 99 classes, and 775 genera were detected in controls. The proportion of phylum Bacteroidetes (56.12%) was lower and that of phylum Firmicutes was higher (37.06%) in CHD patients than those in the controls (60.92% and 32.06%, *P* < 0.05). PCoA and UPGMA tree analysis showed that there were significant differences of gut microbial compositions between the two groups.* Conclusion.* The diversity and compositions of gut flora were different between CHD patients and healthy controls. The incidence of CHD might be associated with the alteration of gut microbiota.

## 1. Introduction

Affecting human health and contributing to risk of disease, human gut microbiota has become a focus of interest from various subjects [1, 2]. The individual composition of gut microbiota has been associated with many factors, including lifestyle, dietary, host genetics, and host metabolism [3–6]. Although the diversity of human gut microbiota has been observed in different individuals, the unique core of its composition is stable, suggesting a potential risk factor of intestinal microbiota for human diseases [7].

Researches have accumulated amount of evidences linking gut microbial compositional alterations and diseases, such as obesity, asthma, type 2 diabetes, arthritis, and cardiovascular disease [8–12]. Relationships between gut microbiota and cardiovascular disease have attracted more and more attentions [13, 14]. Tang and Hazen [15] have reported that the gut microbiota-dependent metabolite, trimethylamine N-oxide, could promote atherosclerosis, whose levels are intensively associated with cardiovascular disease. Oral dietary supplementation with L-carnitine and choline, the precursor of trimethylamine N-oxide by gut microbiota, has been proved to accelerate atherosclerosis in mice [16]. Emoto et al. [17] have demonstrated that alterations of gut microbiota were linked to the incidence of coronary artery disease. However, studies of the gut metagenome, based on 16S ribosomal RNA and high-throughput sequencing on coronary heart disease (CHD), are rare. Thus, more studies are still needed to gain detailed information on variations of gut microbial composition and its impacts on CHD.

To address this, in this study, fecal samples from CHD patients and healthy controls were collected, variable regions of gut bacterial 16S rRNA were amplified, and DNA library was constructed. Then, high-throughput sequencing was used to assess the taxonomic composition of the gut microbiota in CHD patients. The data of this study may provide a theoretical basis of the development and progress of CHD and intestinal flora.

## 2. Materials and Methods

### 2.1. Patients

Between April 2015 and June 2015, 29 CHD in-hospital patients were recruited from Shanghai Renji Hospital and Shanghai Minhang District Central Hospital. Patients were confirmed for CHD by coronary angiography and had undergone the treatment of coronary artery bypass graft or percutaneous coronary intervention. Thirty-five healthy volunteers were recruited from Shanghai Minhang District Chunhui Community as controls. The inclusion criteria were as follows: (i) the subjects had not received antacids, probiotics, antibiotics, or antimicrobial agents within 30 days before sample collection; (ii) there was no organic disease of the digestive system; (iii) they had no gastrointestinal surgery; (iv) there was no history of alcohol abuse, diabetes, or other disease which may affect the intestinal flora; (v) subjects were residents of southern China at the age of 50–85 years. Hypertension was defined as blood pressure > 140/90 mmHg. All subjects gave written informed consent. The Institutional Review Board of the Shanghai Renji Hospital and Shanghai Minhang District Central Hospital approved all study protocols.

### 2.2. Sample Collection and DNA Extraction

Fresh fecal samples (each 2–5 g) were obtained from all the patients under the hospital diet and controls under the usual diet and transferred into sterile collecting pipes. DNA was extracted using the Tiangen stool mini kit (Tiangen, Beijing, China) according to the manufacturer's instructions within 5 hrs after collection. The extracted DNA was stored at −80°C until analysis.

### 2.3. DNA Library Construction and High-Throughput Sequencing

DNA samples were quantified on a Qubit 2.0 Fluorometer (Invitrogen, Carlsbad, CA, USA) and detected under 0.8% agarose gel. A total of 5–50 ng DNA was used to generate amplicons using a MetaVx™ Library Preparation Kit (Genewiz, New Zealand, USA). Three relatively conserved variable regions (V3, V4, and V5) of 16S rRNA were amplified consistent with the previous report [18]. DNA library was verified by Agilent 2100 Bioanalyzer (Agilent Technologies, Palo Alto, CA, USA), quantified using real time PCR (Applied Biosystems, Carlsbad, CA, USA), and multiplexed and loaded on an Illumina MiSeq instrument following the instructions of the manufacturer (Illumina, San Diego, CA, USA). Sequencing was conducted using a 2 × 250 paired-end (PE) configuration, and image analysis and base determination were performed using the MiSeq Control Software on the MiSeq. The initial taxonomy analysis was carried out on Illumina BaseSpace platform.

### 2.4. Data Analysis

Raw data were processed using CASAVA (V1.8.2). Pass Filter Data were obtained after a preliminary analysis of the raw data. Sequences from patients and controls were aligned using Pandaseq (V2.7), spliced according to the end of the overlap, and at least 20 bp overlapping region was retained. In addition, the primer and linker sequences, bases below 20 bp at both ends, and sequences with length less than 400 bp were removed using Trimmomatic (V0.30). Then comparison was performed between splicing sequence and the database using Usearch (V8.0). After the chimera sequence was removed, the final valid sequences were obtained.

Operational taxonomic units (OTUs) were defined at a cutoff of 97% using the RDP rRNA Classifier. Based on Silva database, species classification annotations were performed and species classified information was obtained; then compositions of each sample were calculated at the classification levels of phylum, class, order, family, and genus. Data comparison was from Silva_111 16S rRNA database (https://www.arb-silva.de).

Based on the results of the OTUs analysis, *α* diversity index including Shannon, Chao 1, Ace, and Good's Coverage index were generated using Qiime V1.7 (http://qiime.org/tutorials/otu_picking.html). Then, principal coordinate analysis (PCoA) was performed on the samples and visualized using KiNG Viewer (http://kinemage.biochem.duke.edu/software/king.php). Finally, based on unweighted UniFrac distance matrices, unweighted-pair group method with arithmetic mean (UPGMA) trees were created.

### 2.5. Statistical Analysis

Statistical analyses were performed using the SPSS 19.0 statistical software (SPSS Inc., Chicago, IL, USA). Quantitative variables were expressed as mean ± standard deviation, and qualitative variables were expressed as a percentage. Independent two-sample *t*-test was used for the difference analysis between groups. A *P* value < 0.05 was considered as statistical significance.

## 3. Results

### 3.1. Baseline Characteristics

The mean age of the CHD patients and controls was 68.27 ± 9.54 and 66.14 ± 11.41 years with 51.72% and 51.43% males, respectively (Table 1). There was no significant difference in age, sex, body mass index, and hypertension between the two groups.

### 3.2. High-Throughput Sequencing Data Analysis

A total of 8871390 16S rRNA reads were generated from the 64 fecal samples in the present study, with an average of 305910 ± 59807 reads in CHD patients and 234372 ± 108725 reads in controls. After data optimization, total of 7144415 sequences were obtained from all the fecal samples. The average numbers of sequences in CHD patients and controls were 121312 ± 19293 and 98635 ± 45251, respectively (Table 2). No significant difference in the average number of sequences was found between the two groups.

### 3.3. OTU Analysis

**Figure 1:**
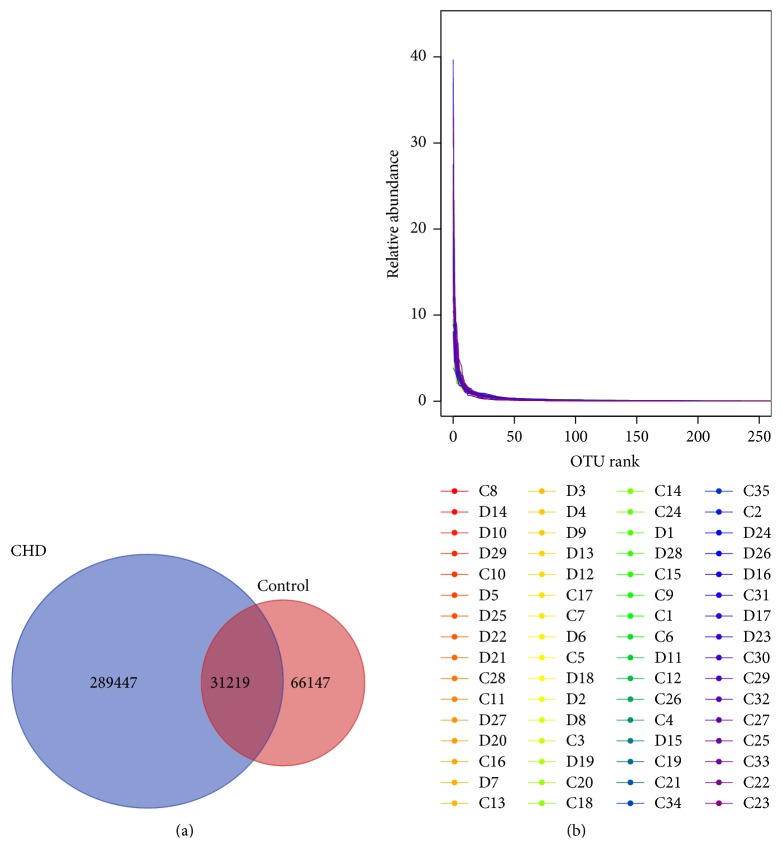
Venn picture (a) and rank-abundance curve (b) of operational taxonomic units (OTUs) in the two groups. The rank-abundance curve was smooth, indicting high evenness among samples. D: CHD patients; C: healthy controls.

OTU analysis showed that total 320666 OTUs and 97366 OTUs were obtained in the CHD and control groups, respectively (Figure 1(a)). Common OTUs in the two groups were 31219. Rank-abundance curve could reflect the abundance and evenness of species. The abundance was reflected by the length of the curve on the horizontal axis and the evenness was reflected by the shape of the curve. If the curve was long and smooth, the abundance and evenness of species were high. After analyzing the rank-abundance curve about OTU of the samples, we found a smooth curve, indicting high evenness among samples (Figure 1(b)).

### 3.4. Species Classification

**Figure 2:**
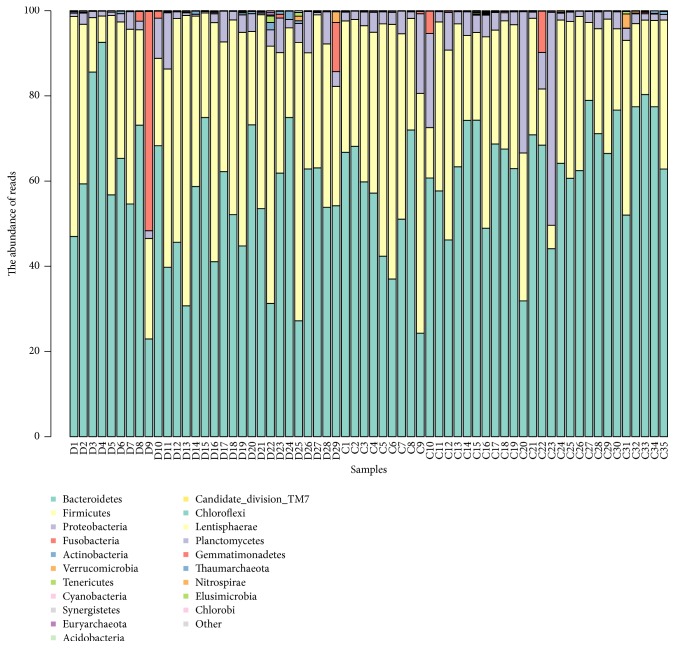
Distribution of relative abundance of top 20 at the phylum level. Each color represents each species. The height of the column represents the abundance of reads. D: CHD patients; C: healthy controls.

Reads corresponding to 38 phyla, 90 classes, and 584 genera were detected in CHD patients' fecal samples. In contrast, 40 phyla, 99 classes, and 775 genera were detected in control samples. There were no significant differences of species classification between these two groups (Figure 2).

### 3.5. Analysis of *α* Diversity Index

As shown in Table 3, *α* diversity of the CHD patients' microbiota was significantly higher than that of the controls. Specially, the Shannon index (7.68 ± 0.81), Chao 1 index (89879.74 ± 27715.57), and Ace index (91473.99 ± 29246.2) in the CHD patients were higher than those in the control group (6.64 ± 0.75, 17797.86 ± 12344.43, and 17964.38 ± 12558.14, resp., *P* < 0.01).

### 3.6. Intestinal Flora Structure Analysis

**Figure 3:**
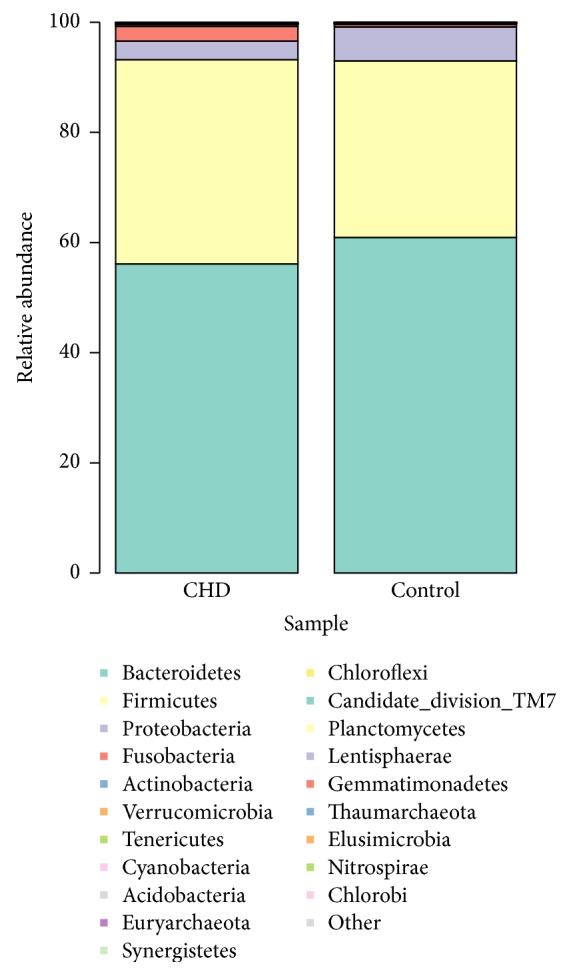
The intestinal flora distribution of total sample on phylum classification in CHD patients and healthy controls.

Sequencing analysis showed that gut microbiota of the two groups were mainly classified into four phyla, including the phyla Bacteroidetes, Firmicutes, Proteobacteria, and Fusobacteria (Figure 3).

**Figure 4:**
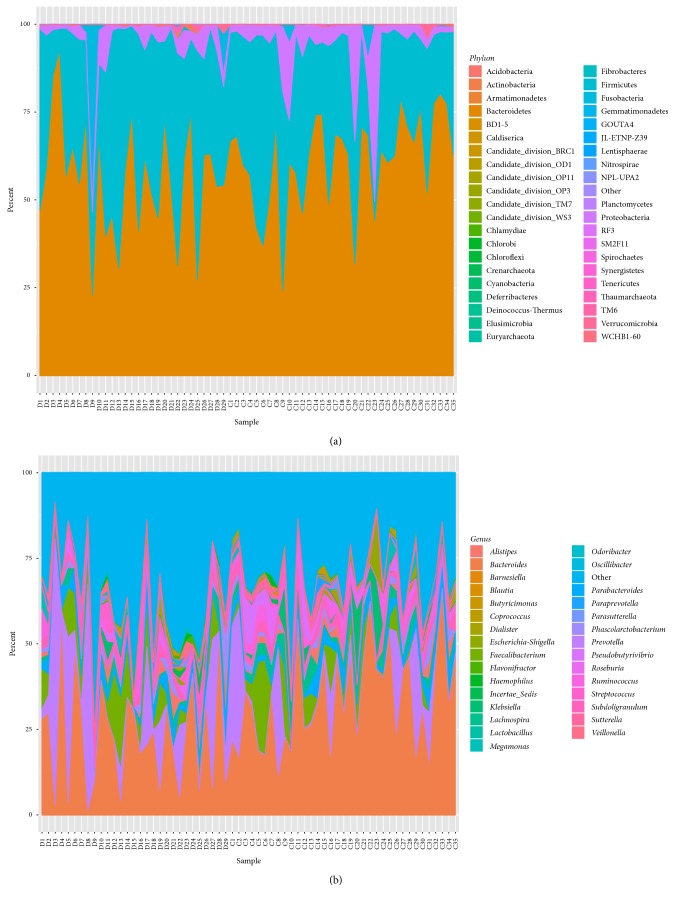
Distribution of species abundance of 64 samples in the phylum (a) and genus (b) classification. Each color respects one species. The height of the columns represents the abundance of reads. D: CHD patients; C: healthy controls.

The phylum Bacteroidetes was found with the highest abundance of reads in CHD patients, accounting for 56.12% in total, which was lower than that in the controls (60.92%, *P* < 0.05, Figure 4(a)). The phylum Firmicutes had the second highest abundance of reads, accounting for 37.06% of total reads in CHD patients, and was higher than that in controls (32.06%, *P* < 0.05, Figure 4(a)). In addition, the phylum Proteobacteria was reduced and the phylum Fusobacteria was increased in CHD patients compared with those in the control group (3.41% versus 6.16% and 2.65% versus 0.45%, resp., Figure 4(a)). The class Bacteroidia, belonging to phylum Bacteroidetes, was significantly decreased in the CHD patient group compared with the control group (56.18% versus 61.35%, *P* < 0.05, Figure 4(b)). The class Clostridia, with the highest abundance of reads in the phylum Firmicutes, accounted for 34.98% of the total reads in CHD patients and was higher than that in the controls (30.60%, *P* < 0.05, Figure 4(b)).

### 3.7. PCoA and UPGMA Tree Analysis

**Figure 5:**
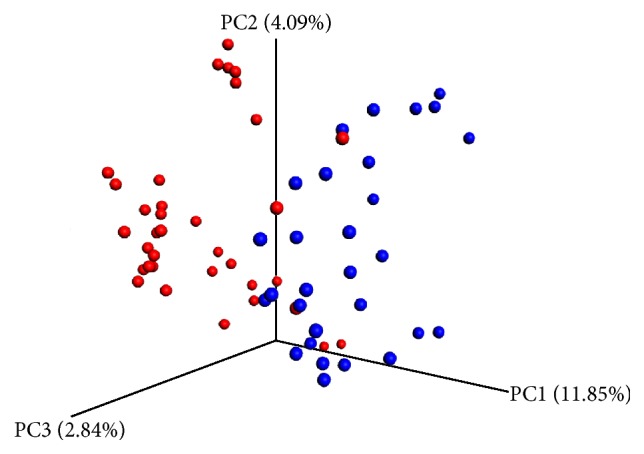
Unweighted UniFrac principal coordinate analysis of fecal microbiota from 64 subjects. Unweighted UniFrac separates the CHD patients (blue) and control microbiota (red).

**Figure 6:**
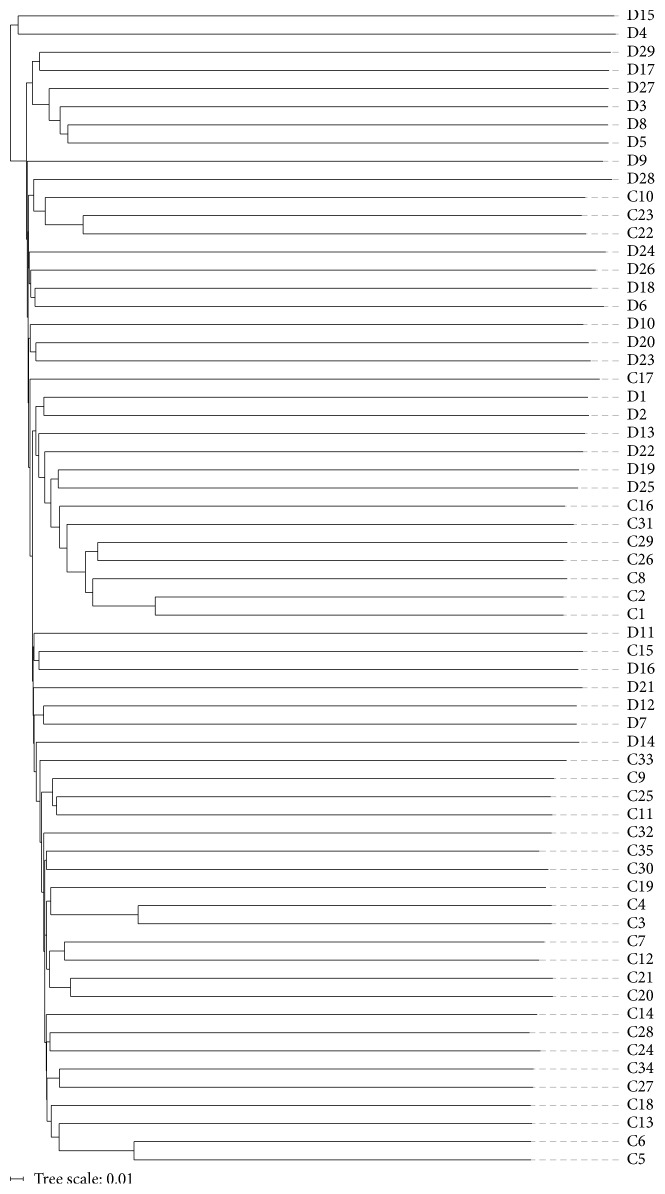
Unweighted-pair group method with arithmetic mean tree of all subjects. D: CHD patients; C: healthy controls.

Based on unweighted UniFrac and Bray-Curtis distance matrices of the 16S rRNA sequences, samples contribution rates of the first PCoA (PC1), second PCoA (PC2), and third PCoA (PC3) were 11.85%, 4.09%, and 2.84%, respectively, which highlighted a clear clustering of the microbial populations of the CHD patients away from that of the controls (Figure 5). This was further confirmed by unweighted UniFrac UPGMA tree (Figure 6).

## 4. Discussion

CHD is a complex multifactorial disease, influenced by numerous genetic and environmental factors. Based on the 16S ribosomal RNA of gut microbiota to carry out high-throughput sequencing, the present study demonstrated that *α* diversity and the gut microbial composition were different between CHD patients and healthy controls.

Diversity is important to maintain ecosystem stability and performance. Microbiota diversity is a new biomarker of health [19]. Loss of gut flora biodiversity is associated with various diseases, including active inflammatory bowel disease, childhood autism, and recurrent* Clostridium* difficile-associated diarrhoea [20–22]. In addition, increased microbiota diversity is linked to an increased health in the elderly [23]. In the *α* diversity analysis (Table 3), our study found that the Shannon index, Chao 1 index, and Ace index were significantly higher in the patients group compared with the control group. This revealed that bacterial communities in case samples had greater genera richness than those in the normal samples. Taken together, our study suggested that increased gut flora diversity might be related to CHD.

Comparison of gut microbial compositions in each group revealed that the phyla Bacteroidetes and Proteobacteria were decreased, whereas the phyla Firmicutes and Fusobacteria were increased in CHD patients compared with the controls. A previous high-throughput sequencing study also demonstrated a decrease in the phylum Bacteroidetes and an increase in the phylum Firmicutes in coronary artery disease [17]. The phylum Bacteroidetes is mainly comprised by two genera,* Bacteroides* and* Prevotella*. The genus* Bacteroides fragilis* plays an important role in mucosal T-cell homeostasis through regulating the function of T-cell [24]. Other* Bacteroides* species establish mutualistic relationships together with the host through providing the biological byproducts necessary for the host and flourishing in the plant polysaccharide-enriched gut surroundings [25]. Additionally,* Bacteroides distasonis*, which is also known as* Parabacteroides distasonis*, is mainly found in the gut of healthy individuals [26]. It has been found to be negatively associated with celiac disease [27] and improving human bowel health release [28]. However, no previous studies have been reported on the role of* Bacteroides* in CHD. More researches are still needed to elaborate this in the future.

Obesity is one of the major risk factors for CHD, as the process from overweight to obese brings in a large number of comorbidities, which are harmful for cardiovascular health [29]. Previous studies have shown that a reduced proportion of the phylum Bacteroidetes and increased proportion of the phylum Firmicutes are associated with obesity, and Firmicutes plays the predominant role [8, 30]. In our study, as there was no difference in body mass index between the two groups, the significant decrease in the proportion of the phylum Bacteroidetes and increase in the proportion of the phylum Firmicutes in the CHD group were possibly reflected by the fact of suffering from CHD.

As an independent marker of the risk of cardiovascular disease, blood Proteobacteria has been verified to be positively related to the onset of cardiovascular complications [31]. By shotgun sequencing of human plasma, Dinakaran et al. [32] have found a parallel reduction in Proteobacteria level in cardiovascular diseases patients in comparison with healthy individuals, which is consistent with findings of our study.

*Fusobacterium nucleatum* is one of the human oral microbiomes. Clinical studies have demonstrated that* Fusobacterium nucleatum* is highly common at the early stages of inflammation implicated in gingivitis [33]. In addition, the accumulation of epidemiologic, clinical, and animal evidence indicates that periodontal infection is a contributing risk factor to heart disease [34]. A previous study has found that specific oral bacterial species are associated with bacteremia and may serve as the etiologic factors for the development of cardiovascular diseases [35]. In our study, Fusobacteria proportion was increased in CHD patients in comparison with the controls, hinting that* Fusobacterium nucleatum* may first cause periodontal disease, which further triggers CHD. However, no studies of the relationships between* Fusobacterium* and CHD have been reported. Thus, more studies are still needed to verify our hypothesis. A recent study has elaborated reductions in the diversity of microbial populations in the obese cohort and also revealed that dietary intervention plays an important role in increasing microbiota diversity in high body mass index individuals [36]. Intestinal microbiota-dependent metabolism of dietary phosphatidylcholine, trimethylamine-N-oxide, is associated with an increase of cardiovascular disease [12, 14]. Diversity in the diet has been found associated with microbiota diversity [23]. In addition, Wu et al. [25] have found that long-term diets could link protein and fat with* Bacteroides* and simple carbohydrates with* Prevotella* to clusters in the gut microbiota. Thus, the differences of gut microbial compositions detected in the current study might be due to the CHD or dietary differences.

This study still had some limitations that should be addressed. First, the samples size of patients was small. Thus, additional larger number of subjects is needed to verify our observations. Second, it is unclear whether the differences of gut flora compositions between groups were a response to CHD or they actively induced the CHD. Our results should be verified by whole genome shotgun sequencing and an epidemiological investigation focusing on the increase in Firmicutes or Fusobacteria and reduction in Bacteroidetes or Proteobacteria.

## 5. Conclusions

In conclusion, the present study suggested that the diversity and composition of gut flora were different between CHD patients and healthy controls. The incidence of CHD may be associated with an alteration of gut microbiota. More studies are needed to illuminate a causal correlation between CHD and gut flora.

## Figures and Tables

**Table 1:** Baseline characteristics of CHD patients and controls.

Variable	CHD patients (*n* = 29)	Controls (*n* = 35)
Age (years)	68.27 ± 9.54	66.14 ± 11.41
Sex (male, %)	15 (51.72)	18 (51.43)
BMI (kg/m^2^)	23.54 ± 3.69	23.70 ± 2.60
Hypertension (%)	86.21	85.71

Mean values (standard deviation) and %  (*n*) represented for continuous and categorical variables, respectively. CHD: coronary heart disease; BMI: body mass index.

**Table 2:** Statistics of pass point data and sequence.

Group	Sample number	Average number of reads	Percentage of HQ bases			N bases%	Number of Sequences		
			Average	Max	Min		Average	Max	Min
CHD	29	305910 ± 59807	68.05 ± 4.06	71.37	60.66	11.2	121312 ± 19293	153195	83869
Control	35	234372 ± 108725	87.00 ± 0.72	88.88	85.85	0.7	98635 ± 45251	341591	64408

CHD: coronary heart disease; HQ bases: high-quality bases with *Q* > 30; N bases: unsure bases.

**Table 3:** Analysis of *α* diversity index between CHD patients and healthy people.

Index	CHD group	Control group
Shannon	7.68 ± 0.81^*∗∗*^	6.64 ± 0.75
Chao 1	89879.74 ± 27715.57^*∗∗*^	17797.86 ± 12344.43
Ace	91473.99 ± 29246.2^*∗∗*^	17964.38 ± 12558.14
Good's Coverage	0.90 ± 0.02^*∗∗*^	0.96 ± 0.01

^*∗∗*^ *P*< 0.01 compared with the control group.

## References

[1] Sommer F., Bäckhed F. (2013). The gut microbiota-masters of host development and physiology. *Nature Reviews Microbiology*.

[2] Sekirov I., Russell S. L., Caetano M Antunes L., Finlay B. B. (2010). Gut microbiota in health and disease. *Physiological Reviews*.

[3] Tremaroli V., Bäckhed F. (2012). Functional interactions between the gut microbiota and host metabolism. *Nature*.

[4] Clarke S. F., Murphy E. F., O'Sullivan O. (2014). Exercise and associated dietary extremes impact on gut microbial diversity. *Gut*.

[5] Conlon M. A., Bird A. R. (2015). The impact of diet and lifestyle on gut microbiota and human health. *Nutrients*.

[6] Goodrich J. K., Waters J. L., Poole A. C. (2014). Human genetics shape the gut microbiome. *Cell*.

[7] Faith J. J., Guruge J. L., Charbonneau M. (2013). The long-term stability of the human gut microbiota. *Science*.

[8] Ley R. E., Turnbaugh P. J., Klein S., Gordon J. I. (2006). Microbial ecology: human gut microbes associated with obesity. *Nature*.

[9] Abrahamsson T. R., Jakobsson H. E., Andersson A. F., Björkstén B., Engstrand L., Jenmalm M. C. (2014). Low gut microbiota diversity in early infancy precedes asthma at school age. *Clinical and Experimental Allergy*.

[10] Qin J., Li Y., Cai Z. (2012). A metagenome-wide association study of gut microbiota in type 2 diabetes. *Nature*.

[11] Scher J. U., Ubeda C., Artacho A. (2015). Decreased bacterial diversity characterizes the altered gut microbiota in patients with psoriatic arthritis, resembling dysbiosis in inflammatory bowel disease. *Arthritis and Rheumatology*.

[12] Tang W. H. W., Wang Z., Levison B. S. (2013). Intestinal microbial metabolism of phosphatidylcholine and cardiovascular risk. *The New England Journal of Medicine*.

[13] Org E., Mehrabian M., Lusis A. J. (2015). Unraveling the environmental and genetic interactions in atherosclerosis: central role of the gut microbiota. *Atherosclerosis*.

[14] Wang Z., Klipfell E., Bennett B. J. (2011). Gut flora metabolism of phosphatidylcholine promotes cardiovascular disease. *Nature*.

[15] Tang W. H. W., Hazen S. L. (2014). The contributory role of gut microbiota in cardiovascular disease. *Journal of Clinical Investigation*.

[16] Koeth R. A., Wang Z., Levison B. S. (2013). Intestinal microbiota metabolism of L-carnitine, a nutrient in red meat, promotes atherosclerosis. *Nature Medicine*.

[17] Emoto T., Yamashita T., Sasaki N. (2016). Analysis of gut microbiota in coronary artery disease patients: a possible link between gut microbiota and coronary artery disease. *Journal of Atherosclerosis and Thrombosis*.

[18] Fu S.-F., Wang F., Shi X.-S., Guo R.-B. (2016). Impacts of microaeration on the anaerobic digestion of corn straw and the microbial community structure. *Chemical Engineering Journal*.

[19] Shanahan F. (2010). Probiotics in perspective. *Gastroenterology*.

[20] Kang D.-W., Park J. G., Ilhan Z. E. (2013). Reduced incidence of Prevotella and other fermenters in intestinal microflora of autistic children. *PLoS ONE*.

[21] Ott S. J., Musfeldt M., Wenderoth D. F. (2004). Reduction in diversity of the colonic mucosa associated bacterial microflora in patients with active inflammatory bowel disease. *Gut*.

[22] Chang J. Y., Antonopoulos D. A., Kalra A. (2008). Decreased diversity of the fecal microbiome in recurrent *Clostridium difficile*-associated diarrhea. *Journal of Infectious Diseases*.

[23] Claesson M. J., Jeffery I. B., Conde S. (2012). Gut microbiota composition correlates with diet and health in the elderly. *Nature*.

[24] Mazmanian S. K., Round J. L., Kasper D. L. (2008). A microbial symbiosis factor prevents intestinal inflammatory disease. *Nature*.

[25] Wu G. D., Chen J., Hoffmann C. (2011). Linking long-term dietary patterns with gut microbial enterotypes. *Science*.

[26] Xu J., Mahowald M. A., Ley R. E. (2007). Evolution of symbiotic bacteria in the distal human intestine. *PLoS Biology*.

[27] Sánchez E., Donat E., Ribes-Koninckx C., Calabuig M., Sanz Y. (2010). Intestinal Bacteroides species associated with coeliac disease. *Journal of Clinical Pathology*.

[28] Clarke J. M., Topping D. L., Christophersen C. T. (2011). Butyrate esterified to starch is released in the human gastrointestinal tract. *The American Journal of Clinical Nutrition*.

[29] Poirier P., Giles T. D., Bray G. A. (2006). Obesity and cardiovascular disease: pathophysiology, evaluation, and effect of weight loss: an update of the 1997 American Heart Association Scientific Statement on obesity and heart disease from the Obesity Committee of the Council on Nutrition, Physical Activity, and Metabolism. *Circulation*.

[30] Furet J.-P., Kong L.-C., Tap J. (2010). Differential adaptation of human gut microbiota to bariatric surgery-induced weight loss: links with metabolic and low-grade inflammation markers. *Diabetes*.

[31] Amar J., Lange C., Payros G. (2013). Blood microbiota dysbiosis is associated with the onset of cardiovascular events in a large general population: the D.E.S.I.R. study. *PLoS ONE*.

[32] Dinakaran V., Rathinavel A., Pushpanathan M., Sivakumar R., Gunasekaran P., Rajendhran J. (2014). Elevated levels of circulating DNA in cardiovascular disease patients: metagenomic profiling of microbiome in the circulation. *PLoS ONE*.

[33] Moore W. E. C., Moore L. V. H. (1994). The bacteria of periodontal diseases. *Periodontology 2000*.

[34] Genco R., Offenbacher S., Beck J. (2002). Periodontal disease and cardiovascular disease: epidemiology and possible mechanisms. *The Journal of the American Dental Association*.

[35] Nakano K., Nemoto H., Nomura R. (2009). Detection of oral bacteria in cardiovascular specimens. *Oral Microbiology and Immunology*.

[36] Cotillard A., Kennedy S. P., Kong L. C. (2013). Dietary intervention impact on gut microbial gene richness. *Nature*.

